# Development of antibiotic treatment algorithms based on Gram stain to restrict use of broad-spectrum antibiotics in the treatment of ventilator-associated pneumonia: a retrospective analysis

**DOI:** 10.1186/cc14178

**Published:** 2015-03-16

**Authors:** J Yoshimura, T Kiguchi, A Matsushima, S Fujimi

**Affiliations:** 1 Osaka General Medical Center, Osaka, Japan

## Introduction

Ventilator-associated pneumonia (VAP) is a common and serious problem in ICUs. Several studies have been conducted to determine the effectiveness of Gram stain of tracheal aspirates for diagnosing VAP. However, the effectiveness for predicting causative microorganisms and guiding appropriate initial antibiotic therapy has not been evaluated. The purpose of this study is to determine whether Gram stain of tracheal aspirates can guide appropriate initial antibiotic therapy for VAP.

## Methods

We retrospectively assessed two hypothetical empirical antibiotic treatment algorithms for VAP on an 18-bed ICU. Data on consecutive episodes of microbiologically confirmed VAP were collected over a period of 22 months and divided into a derivation (1 February 2013 to 30 November 2013) and validation (1 December 2013 until 15 November 2014) cohort. We constructed two algorithms in the derivation cohort. One is a local ecology-based algorithm (LEBA), according to clinical risk factors for MDR and susceptibility results in our hospital. The other is a Gram stain-based algorithm (GSBA). The selection of antibiotics on GSBA was directed against pathogens predicted from the results of bedside Gram staining of tracheal aspirates collected just before antibiotic therapy. Subsequently, LEBA and GSBA were retrospectively reviewed and compared with actually prescribed antibiotics in the validation cohort.

## Results

The first 50 VAP episodes made up the derivation cohort and the subsequent 50 VAP episodes the validation cohort. Antibiotic coverage rates by applying LEBA and GSBA were identical (96% vs. 96%). GSBA proposed more narrow spectrum therapy as compared with LEBA (*P *< 0.001). GSBA recommended carbapenems in significantly less episodes than LEBA (*P *< 0.001) and the same episodes as actually prescribed initial therapy (*P *= 1). However, there was significant increase of antibiotic coverage rates in GSBA compared with the actually prescribed initial therapy (96% vs. 78%, *P *= 0.015).

## Conclusion

Antibiotic coverage rates on GSBA were comparable with LEBA. The use of GSBA would result in a significant reduction of the administration of broad-spectrum antibiotics. Bedside Gram staining may be useful to guide appropriate initial antibiotic therapy for VAP.

